# Supplementary material for: Splice-Junction-Based Mapping of Alternative Isoforms in the Human Proteome
Source: Cell Rep. Author manuscript; Available in PMC 2020 Jan 15. (PMC6961840; doi:10.1016/j.celrep.2019.11.026)
Supplement: 3 [file NIHMS1546469-supplement-3.zip › DF2/PXD009737/TestisGluC-235-Q5VT52-ALTSTNPKAALKSKIVAE.pdf]

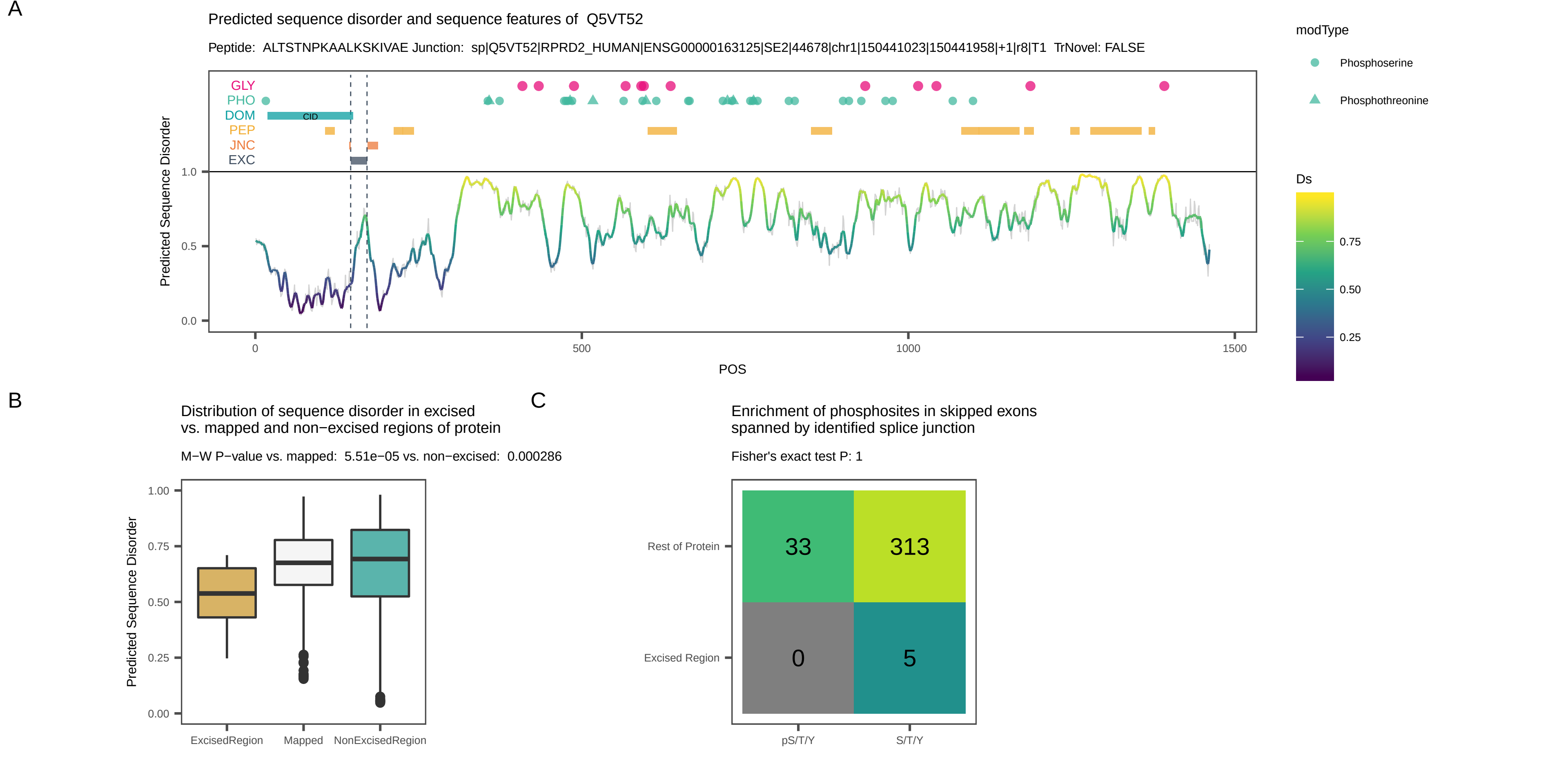
A
Predicted sequence disorder and sequence features of Q5VT52
Peptide: ALTSTNPKAALKSKIVAE Junction: sp|Q5VT52|RPRD2_HUMAN|ENSG00000163125|SE2|44678|chr1|150441023|150441958|+1|r8|T1 TrNovel: FALSE
GLY
PHO
DOM
CID
PEP
JNC
EXC
Predicted Sequence Disorder
1.0
0.5
0.0
0
500
1000
1500
POS
modType
Phosphoserine
Phosphothreonine
Ds
0.75
0.50
0.25
B
Distribution of sequence disorder in excised
vs. mapped and non−excised regions of protein
M−W P−value vs. mapped: 5.51e−05 vs. non−excised: 0.000286
Predicted Sequence Disorder
1.00
0.75
0.50
0.25
0.00
ExcisedRegion
Mapped
NonExcisedRegion
C
Enrichment of phosphosites in skipped exons
spanned by identified splice junction
Fisher's exact test P: 1
Rest of Protein
Excised Region
33
313
0
5
pS/T/Y
S/T/Y
